# To propose adding index of achievement (IOA) to IMRT QA process

**DOI:** 10.1186/s13014-018-1055-5

**Published:** 2018-06-15

**Authors:** Dong-Su Kim, Siyong Kim, Seong-Hee Kang, Tae-Ho Kim, So-Hyun Park, Kyeong-Hyeon Kim, Min-Seok Cho, Dong-Seok Shin, Yu-Yun Noh, Jin-Beom Chung, Tae Suk Suh

**Affiliations:** 1 Department of Biomedical Engineering and Research Institute of Biomedical Engineering, College of Medicine, The Catholic University of Korea, 222. Banpo-daero, Seocho-gu, Seoul, 06591 South Korea; 2 Department of Radiation Oncology, Virginia Commonwealth University, 401 College Street, Richmond, VA 23298-0058 USA; 3 Department of Radiation Oncology, Seoul National University Bundang Hospital, Gumi-ro 173 beon-gil, Bundang-gu, Seongnam-si, Gyeonggi-do 13620 South Korea; 4 Department of Radiation Oncology, Jeju National University Hospital, 15, Aran 13-gil, Jeju-si, Jeju-do 63241 South Korea; 5 Department of Radiation Oncology, Asan Medical Center, 88, Olympic-ro 43-gil, Songpa-gu, Seoul, 05505 South Korea; 6 Department of Radiation Oncology, Eulji University Hospital, 95, Dunsanseo-ro, Seo-gu, Daejeon, 35233 South Korea

**Keywords:** IMRT, IMRT QA, Patient specific QA, QA index

## Abstract

**Background:**

In intensity modulated radiation therapy (IMRT) quality assurance (QA), evaluation of QA result using a pass/non-pass strategy under an acceptance criterion often suffers from lack of information on how good the plan is in absolute manner. In this study, we suggested adding an index system, previously developed for dose painting technique, to current IMRT QA process for better understanding of QA result.

**Methods:**

The index system consists of three indices, index of achievement (IOA), index of hotness (IOH) and index of coldness (IOC). As indicated by its name, IOA does measure the level of agreement. IOH and IOC, on the other hand, measure the magnitude of overdose and underdose, respectively. A systematic analysis was performed with three 1-dimensional hypothetical dose distributions to investigate the characteristics of the index system. The feasibility of the system was also assessed with clinical volumetric modulated arc therapy (VMAT) QA cases from 8 head & neck and 5 prostate patients. In both simulation studies, certain amount of errors was intentionally induced to each dose distribution. Furthermore, we applied the proposed system to compare calculated with actual measured data for a total of 60 patients (30 head & neck and 30 prostate cases). QA analysis was made using both the index system and gamma method, and results were compared.

**Results:**

While the gamma evaluation showed limited sensitivity in evaluating QA result depending on the level of tolerance criteria used, the proposed indices tended to better distinguish plans in terms of the amount of errors. Hotness and coldness of prescribed dose in the plan could be evaluated quantitatively by the indices.

**Conclusions:**

The proposed index system provides information with which IMRT QA result would be better evaluated, especially when gamma pass rates are identical or similar among multiple plans. In addition, the independency of the index system on acceptance criteria would help making clear communications among readers of published articles and researchers in multi-institutional studies.

## Background

Patient specific quality assurance (QA) in intensity modulated radiation therapy (IMRT) is important to verify the accuracy of dose calculation and delivery. IMRT QA is commonly accomplished by comparing a calculated dose distribution with an actually measured dose distribution [1, 2].

Many reports have been published with regard to quantifiable indices for IMRT QA evaluation and the assessment of their performances in various situations [2–10]. Presently, a well-accepted approach is to count how many measurement points are within a preset criterion [3, 4, 11]. Most criteria are made based on either dose difference (DD) or distance-to-agreement (DTA), or both. The gamma index is similar in principle, but it does utilize a criterion that combines both dose difference and DTA into a single parameter [12]. There are clear advantages in the gamma method. Obviously, dealing with one quantity (i.e., gamma-index) is more straightforward than doing with two quantities. In addition, evaluating whether an IMRT QA satisfies or not based on the number of passing points under the given criteria is simple and convenient in certain aspect thus, the gamma index method has been preferably adopted in many clinic sites.

In the pass rate-based approach, however, it is difficult to estimate the absolute matching quality of each plan between measurement and calculation because its pass rate can vary significantly depending on how the acceptance criterion is chosen [13–15]. Such issue can be problematic when a reader/reviewer is trying to understand the matching quality of plans reported in publications and/or submitted for review in multi-institutional clinical trials. To be specific, for instance, in case institution A requires over 90% pass rate under a DD/DTA criterion of 2%/2 mm and institution B does over 95% pass rate under a DD/DTA criterion of 3%/3 mm, it is not easy to judge which institution does keep higher QA result overall. Therefore, it would be beneficial to have an additional system that simply supplements the current method by providing the matching quality of IMRT plans independent of the preset criteria.

Recently, index of achievement (IOA) system, a plan quality evaluation approach under the dose-painting paradigm, has been introduced that takes point-by-point relative dose differences into account to provide simple indices [16]. The concept of IOA is basically based on the first principle of direct dose difference thus, simple but effective in the dose-painting strategy for which typical homogeneity index, a popular strategy in conventional therapy, does not work at all.

While IOA method was developed for plan evaluation in planning stage, we believe, it can be utilized for QA stage as well. Only difference is that comparison is made between measured dose and planned dose in QA stage instead of between planned dose and prescribed dose. Therefore, in this study, we tried to apply IOA method to QA evaluation in IMRT QA and investigated its feasibility for compensating the limitations of the current method. There are two more indices in the IOA approach, index of hotness (IOH) and index of coldness (IOC), and they are also included to measure the overall levels of overdose and underdose. For both hypothetical dose profiles and actual IMRT planning dose distributions, the characteristics of obtained indexing values were analyzed and compared with that of gamma evaluation.

## Methods

### Formula

Three indices in this approach (IOA, IOH and IOC) are expressed as follows:

1 $$ IOA=1+\sqrt{\sum \limits_i\left[{\left(\frac{D_{eva,i}-{D}_{ref,i}}{D_{norm}}\right)}^2\times \frac{1}{N}\times {B}_i\right]} $$

2 $$ IOH=1+\sqrt{\sum \limits_i\left[{\left(\frac{D_{eva,i}-{D}_{ref,i}}{D_{norm}}\right)}^2\times \frac{1}{N}\times {B}_i\right]},\left(\mathrm{only}\ \mathrm{for}\ {D}_{eva,i}>{D}_{ref,i}\right) $$

3 $$ IOC=1-\sqrt{\sum \limits_i\left[{\left(\frac{D_{eva,i}-{D}_{ref,i}}{D_{norm}}\right)}^2\times \frac{1}{N}\times {B}_i\right]},\left(\mathrm{only}\ \mathrm{for}\ {D}_{eva,i}<{D}_{ref,i}\right) $$

where,

4 $$ {D}_{norm}=\left\{\begin{array}{ll}{D}_{ref,\max },& \mathrm{for}\kern0.5em \mathrm{global}\kern0.5em \mathrm{normalization}\\ {}{D}_{ref,i},& \mathrm{for}\kern0.5em \mathrm{local}\kern0.5em \mathrm{normalization}\end{array}\right. $$

and, *D*_*ref*, *i*_ and *D*_*eva*, *i*_are the reference and evaluation dose of *i*th voxel, *N* is the total number of voxels, *B*_*i*_ is the binary factor of *i*th voxel allowing for binary selection of each voxel in calculating an index, and *D*_*ref*, max_ is the maximum of the reference doses, respectively. Regarding *B*_*i*_, we used three sub-binary factors, *b*_*i*, *TH*_, *b*_*i*, *H*_ and *b*_*i*, *C*_, each to take into account the threshold of dose, hotness and coldness, respectively. *B*_*i*_ can be a product of several *b*_*i*_ values and each of them corresponds to a specific condition. Now, Eq. (1), (2), and (3) can be rewritten as described below:

5 $$ {IOA}_{TH}=1+\sqrt{\sum \limits_i\left[{\left(\frac{D_{eva,i}-{D}_{ref,i}}{D_{norm}}\right)}^2\times \frac{1}{N_{TH}}\times {b}_{i, TH}\right]} $$

6 $$ {IOH}_{TH}=1+\sqrt{\sum \limits_i\left[{\left(\frac{D_{eva,i}-{D}_{ref,i}}{D_{norm}}\right)}^2\times \frac{1}{N_{TH}}\times {b}_{i, TH}\times {b}_{i,H}\right]} $$

7 $$ {IOC}_{TH}=1-\sqrt{\sum \limits_i\left[{\left(\frac{D_{eva,i}-{D}_{ref,i}}{D_{norm}}\right)}^2\times \frac{1}{N_{TH}}\times {b}_{i, TH}\times {b}_{i,C}\right]} $$

where,

8 $$ {b}_{i, TH}=\left\{\begin{array}{ll}1,& {D}_{ref,i}\ge {D}_{ref, TH}\\ {}0,& \mathrm{otherwise}\end{array}\right. $$

9 $$ {b}_{i,H}=\left\{\begin{array}{ll}1,& {D}_{eva,i}>{D}_{ref,i}\\ {}0,& \mathrm{otherwise}\end{array}\right. $$

10 $$ {b}_{i,C}=\left\{\begin{array}{ll}1,& {D}_{eva,i}<{D}_{ref,i}\\ {}0,& \mathrm{otherwise}\end{array}\right. $$

and, *D*_*ref*, *TH*_ is the threshold dose which is ‘*TH*’% dose of the maximum reference dose and *N*_*TH*_ is the number of voxels having dose not smaller than *D*_*ref*, *TH*_. Note that each index is expressed with a subscript, ‘*TH*’ added to indicate voxels having lower than ‘*TH*’% of maximum dose in the reference plan are excluded in index calculation. As defined, in addition, IOH is obtained in the region where evaluation doses are higher than reference doses and IOC is opposite.

It is clear that each index is a single value and becomes ‘1’ in an ideal case, that is, when the evaluation dose distribution perfectly matches with the reference. Note that for non-ideal cases, the value of IOC is always smaller than ‘1’ while those of IOA and IOH are larger than ‘1’.

The index system performs a point-by-point calculation on the identical grid point (i.e., indicated as *i*th voxel) of both the reference and evaluation distribution. Intuitively, the total number of voxels to be evaluated (i.e., the whole domain of *i*th voxel) better be determined by whichever distribution between the reference and evaluation has smaller number of available points. However, when the locations of available points do not exactly match between the reference and evaluation distributions, interpolations can be made to generate values on certain grid points and index calculations can be performed based on such grid points, which is what used in this study.

### Systematic study with 1-D hypothetical dose distribution

**Fig. 1:**
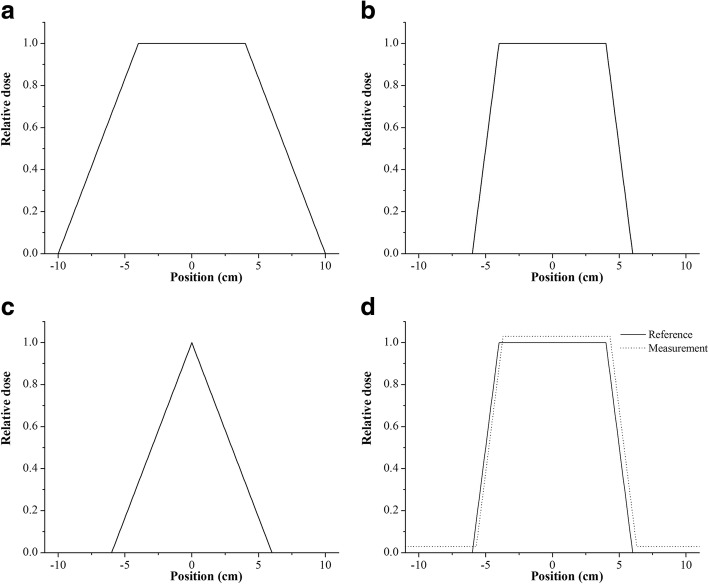
Hypothetical 1-dimensional dose model profiles used for systematic analysis: (**a**) model A, (**b**) model B, (**c**) model C, and (**d**) model B as a reference (solid plot) and a simulated dose profile with 3%/3 mm displacement from the reference (dotted plot)

A systematic analysis was performed on three one-dimensional (1-D) normalized dose distributions using MATLAB (The MathWorks, Inc., Natick, MA) as shown in Fig. 1 (a, b and c). As can be seen, (a) and (b) have the same size of flat region but different penumbra while (a) and (c) have the same penumbra but different size of flat region. For convenience we will call dose distributions (a), (b) and (c) as Model A, Model B and Model C, respectively. For the systematic analysis, simulated evaluation distributions were generated from the reference distributions by modifying them in both magnitude and location. For magnitude change, 0 to 3% of maximum reference dose at 1% interval were added. On the other hand, for location change, lateral displacements by 0 to 3 mm at 1 mm interval were made. The resolution used for Model A, B, and C was 1 mm. Figure 1 (d) illustrates a simulated evaluated dose distribution (dotted plot) together with the reference of Model B (solid plot) that contains uncertainties of + 3% of maximum reference dose in magnitude and + 3 mm in location. For each simulated case, IOA was calculated and compared with gamma evaluation results under 1%/1 mm, 2%/2 mm, 3%/3 mm and 4%/4 mm DD/DTA acceptance criteria for both global and local normalization.

### 2-D systematic study using clinical cases

In order to investigate the feasibility of the proposed indices, a total of 13 (8 head & neck and 5 prostate) clinical volumetric modulated arc therapy (VMAT) QA cases were considered with IRB (Institutional Review Board) approval (Catholic Medical Center Protocol ID #KC16RISI0537). Dose calculations were performed with the Eclipse portal dose image prediction (PDIP, Varian Medical System, Palo Alto, CA). Calculated dose distributions were in 40 × 30 cm^2^ size consisting of 512 × 384 pixels with 0.734 mm pixel pitch.

Similar to the 1-D study, using MATLAB, calculated dose distributions were modified by intentionally adding errors (ranging from − 3 to + 3% of maximum reference dose with 1% interval in magnitude and − 3 mm to + 3 mm with 1 mm interval in either lateral- or longitudinal-direction) to generate a total of 96 erroneous dose distributions per each case (i.e., a total of 1248 simulations). For each simulation, dose distributions were interpolated to 1 mm grid size to easily apply intentional spatial errors in the interval of 1 mm. In condition of both global and local normalizations, all of 3 indices (i.e., IOA, IOH and IOC) were obtained and compared with gamma evaluation results. Regarding gamma evaluation, 4 different DD/DTA criteria (1%/1 mm, 2%/2 mm, 3%/3 mm and 4%/4 mm) were considered (i.e., a total of 4992 gamma evaluations). As commonly adopted, a threshold of 10% of the maximum dose was applied in this study.

**Fig. 2:**
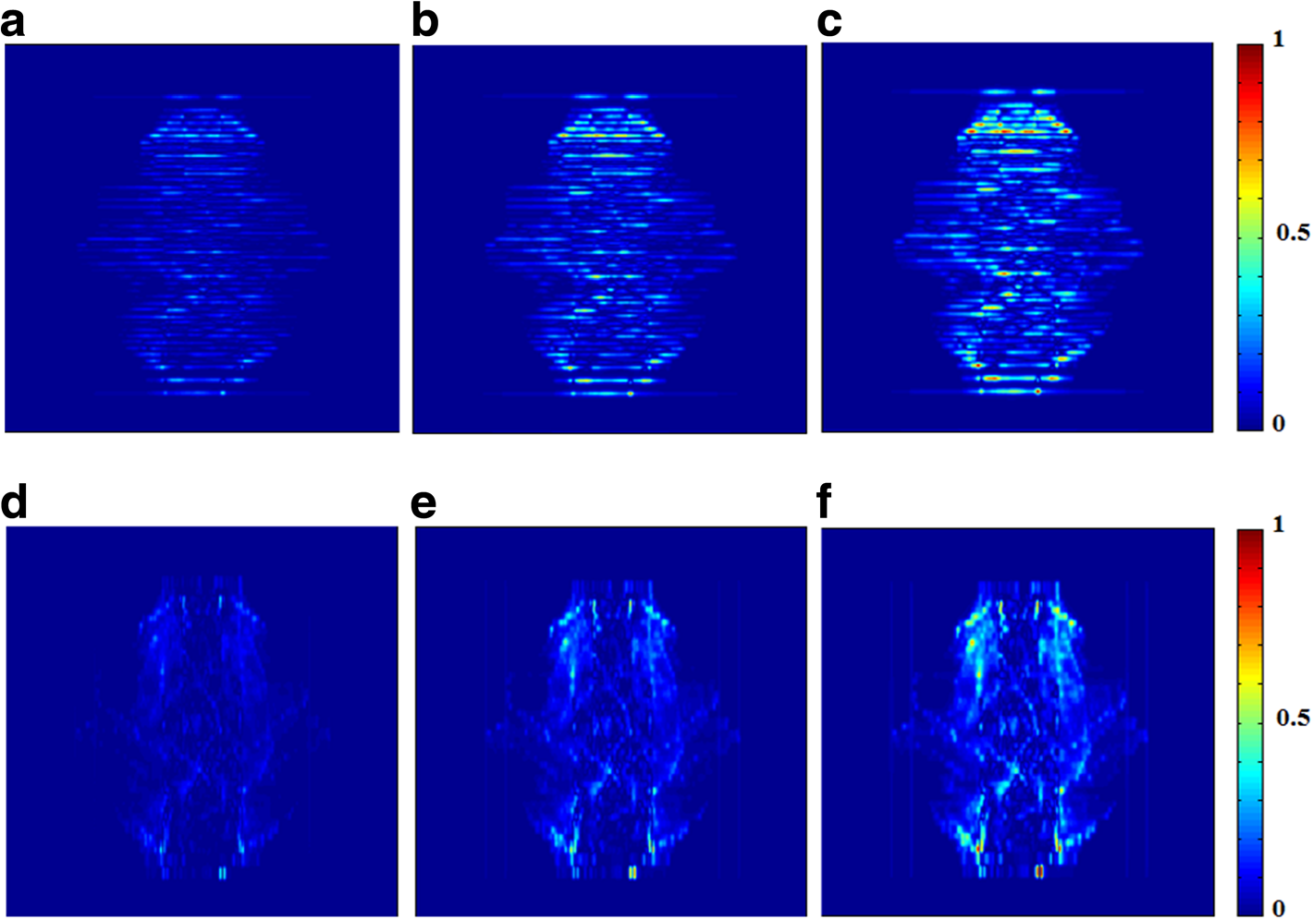
Examples of normalized dose difference for one of the head and neck cases when intentional spatial displacements are applied, from 1 to 3 mm along (**a** - **c**) horizontal or (**d** – **f**) longitudinal direction, respectively. All values were normalized to the maximum dose difference among the same group [i.e., (**a** – **c**) horizontal group or (**d** – **f**) longitudinal group]

Figure 2 shows examples of normalized dose difference for one of the head and neck cases when intentional spatial displacements are applied, from 1 to 3 mm along (a - c) horizontal or (d – f) longitudinal direction, respectively. All values were normalized to the maximum dose difference among the same group [i.e., (a – c) horizontal group or (d – f) longitudinal group].

### Application for comparing calculated with measured data for clinical cases

The proposed method was applied to a total of 60 cases (30 from head & neck and another 30 from prostate patients) under IRB approval (Seoul National Bundang Hospital Protocol ID #B-1711-432-108). The calculated and measured data were based on VMAT QA cases using the PDIP and electronic portal imaging device (EPID) dosimetry. Acquisition conditions for calculated and measured data are shown in Table 1. For each case, the calculated and measured dose distributions were interpolated to 1 mm grid size for consistent evaluation regardless of acquisition conditions. IOA values in both global and local normalizations were obtained and compared with gamma evaluation results. Regarding gamma evaluation, 4 different DD/DTA criteria (1%/1 mm, 2%/2 mm, 3%/3 mm and 4%/4 mm) were considered. Also, a threshold of 10% of the maximum dose was applied in this study.

**Table 1:** Acquisition conditions for calculated and measured dose distribution

Active area (cm × cm)	40 × 40	40 × 30
Calculated matrix / Pixel pitch (mm)	1024 × 1024 / 0.393	512 × 384 / 0.786
Measured matrix / Pixel pitch (mm)	1190 × 1190 / 0.336	1024 × 768 / 0.392
EPID model	a-Si 1200	a-Si 1000
Head and Neck (cases)	21	9
Prostate (cases)	13	17

## Results

### Systematic study with 1-D hypothetical dose distribution

Table 2 shows the result of 1-D systematic study (i.e., IOA values and gamma pass rate under 1%/1 mm, 2%/2 mm, 3%/3 mm and 4%/4 mm acceptance criteria for global normalization). Just for convenience, each case was ranked based on IOA (i.e., in the order of achievement) within the group it belongs to (i.e., starting with ‘1’ for the best case). In the results of IOA, the values varied through most cases and showed a trend of gradual increase with the amount of error, demonstrating strong distinguishability of QA results. Contrary to the IOA analysis, every simulated gamma analysis cases up to 2%/2 mm intended error showed 100% pass rate under 3%/3 mm criterion for all of 3 dose distributions. Simulations of 3%/0 mm and 0%/3 mm also showed 100% gamma pass rate.

**Table 2:** The 1-D systematic study results of the gamma method and the IOA for the global normalization

	Intentional Dose Error =>		0%				+ 1%				+ 2%				+ 3%		
Intentional Spatial Error	Model =>		A	B	C		A	B	C		A	B	C		A	B	C
0 mm	Gamma pass	4%/4 mm	100.0	100.0	100.0		100.0	100.0	100.0		100.0	100.0	100.0		100.0	100.0	100.0
		3%/3 mm	100.0	100.0	100.0		100.0	100.0	100.0		100.0	100.0	100.0		100.0	100.0	100.0
	rate (%)	2%/2 mm	100.0	100.0	100.0		100.0	100.0	100.0		100.0	100.0	100.0		56.9	32.1	90.8
		1%/1 mm	100.0	100.0	100.0		100.0	100.0	100.0		0	30.5	0		0	30.5	0
	IOA	Value	1.000	1.000	1.000		1.010	1.010	1.010		1.020	1.020	1.020		1.030	1.030	1.030
		Rank	1	1	1		2	2	2		5	3	5		9	6	7
1 mm	Gamma pass	4%/4 mm	100.0	100.0	100.0		100.0	100.0	100.0		100.0	100.0	100.0		100.0	100.0	100.0
		3%/3 mm	100.0	100.0	100.0		100.0	100.0	100.0		100.0	100.0	100.0		99.5	99.2	99.2
	rate (%)	2%/2 mm	100.0	100.0	100.0		100.0	100.0	100.0		71.6	99.2	54.2		28.9	32.1	45.8
		1%/1 mm	100.0	100.0	100.0		71.6	84.7	54.2		28.4	16.0	45.8		28.0	16.0	45.0
	IOA	Value	1.013	1.028	1.016		1.016	1.029	1.019		1.024	1.034	1.026		1.033	1.041	1.034
		Rank	3	4	3		4	5	4		6	7	6		11	8	10
2 mm	Gamma pass	4%/4 mm	100.0	100.0	100.0		100.0	100.0	100.0		100.0	100.0	100.0		100.0	100.0	100.0
		3%/3 mm	100.0	100.0	100.0		100.0	100.0	100.0		100.0	100.0	100.0		71.1	98.5	54.2
	rate (%)	2%/2 mm	100.0	100.0	100.0		71.6	84.7	55.0		71.1	84.0	54.2		29.4	17.6	46.6
		1%/1 mm	43.1	69.5	9.2		43.1	68.7	9.2		28.9	1.5	45.8		28.4	1.5	45.0
	IOA	Value	1.025	1.055	1.032		1.027	1.056	1.033		1.032	1.058	1.038		1.039	1.062	1.044
		Rank	7	9	8		8	10	9		10	11	11		14	12	12
3 mm	Gamma pass	4%/4 mm	100.0	100.0	100.0		100.0	100.0	100.0		100.0	100.0	100.0		71.6	99.2	55.7
		3%/3 mm	100.0	100.0	100.0		72.0	84.7	55.7		71.6	84.7	55.7		70.6	83.2	54.2
	rate (%)	2%/2 mm	44.1	69.5	10.7		43.1	68.7	9.9		70.6	67.7	53.4		29.9	3.8	46.6
		1%/1 mm	42.2	67.9	9.2		42.2	67.2	8.4		1.4	1.5	1.5		1.4	1.5	1.5
	IOA	Value	1.037	1.081	1.047		1.039	1.082	1.048		1.042	1.084	1.051		1.048	1.086	1.056
		Rank	12	13	13		13	14	14		15	15	15		16	16	16

In the local normalization, as shown in Table 3, most cases showed 100% pass rate under such condition except for cases having 2% or more dose error from model A and C. Obviously, therefore, gamma method is not able to distinguish each simulated case from another in terms of its quality in such situations. For other cases, pass rate varied from ~ 99 to 0%, showing certain level of discernment ability when the amount of error is relatively large.

**Table 3:** The 1-D systematic study results of the gamma method and the IOA for the local normalization

	Intentional Dose Error =>		0%				+ 1%				+ 2%				+ 3%		
Intentional Spatial Error	Model =>		A	B	C		A	B	C		A	B	C		A	B	C
0 mm	Gamma pass	4%/4 mm	100.0	100.0	100.0		100.0	100.0	100.0		99.0	100.0	98.3		99.0	100.0	98.3
		3%/3 mm	100.0	100.0	100.0		100.0	100.0	100.0		99.0	100.0	98.3		99.0	100.0	98.3
	rate (%)	2%/2 mm	100.0	100.0	100.0		100.0	100.0	100.0		99.0	100.0	98.3		59.3	35.3	98.3
		1%/1 mm	100.0	100.0	100.0		100.0	100.0	100.0		0	33.6	0		0	33.6	0
	IOA	Value	1.000	1.000	1.000		1.077	1.034	1.099		1.154	1.068	1.199		1.231	1.101	1.298
		Rank	1	1	1		2	2	2		5	3	5		8	4	8
1 mm	Gamma pass	4%/4 mm	100.0	100.0	100.0		100.0	100.0	100.0		99.0	100.0	98.3		99.0	100.0	98.3
		3%/3 mm	100.0	100.0	100.0		100.0	100.0	100.0		99.0	100.0	98.3		99.0	100.0	98.3
	rate (%)	2%/2 mm	100.0	100.0	100.0		100.0	100.0	100.0		69.8	100.0	49.6		30.2	35.3	49.6
		1%/1 mm	100.0	100.0	100.0		70.4	84.0	50.4		29.6	17.6	49.6		29.6	17.6	49.6
	IOA	Value	1.128	1.164	1.165		1.149	1.167	1.193		1.200	1.177	1.258		1.264	1.192	1.341
		Rank	3	5	3		4	6	4		6	7	6		10	8	10
2 mm	Gamma pass	4%/4 mm	100.0	100.0	100.0		100.0	100.0	100.0		99.0	100.0	98.3		99.0	100.0	98.3
		3%/3 mm	100.0	100.0	100.0		100.0	100.0	100.0		72.9	100.0	54.6		69.8	100.0	50.4
	rate (%)	2%/2 mm	100.0	100.0	100.0		70.4	84.0	51.3		69.8	84.0	50.4		30.7	19.3	50.4
		1%/1 mm	40.2	67.2	0.8		40.2	67.2	0.8		30.2	1.7	49.6		30.2	1.7	49.6
	IOA	Value	1.225	1.286	1.290		1.250	1.294	1.323		1.294	1.305	1.379		1.349	1.320	1.451
		Rank	7	9	7		9	10	9		11	11	11		14	12	14
3 mm	Gamma pass	4%/4 mm	100.0	100.0	100.0		100.0	100.0	100.0		77.4	100.0	62.2		69.8	100.0	51.3
		3%/3 mm	100.0	100.0	100.0		70.9	84.0	52.1		69.8	84.0	51.3		69.8	84.0	51.3
	rate (%)	2%/2 mm	40.7	67.2	1.7		40.2	67.2	1.7		69.8	67.2	50.4		31.2	3.4	50.4
		1%/1 mm	39.7	66.4	1.7		39.7	66.4	0.8		1.0	1.7	0.8		1.0	1.7	0.8
	IOA	Value	1.318	1.404	1.411		1.347	1.415	1.448		1.389	1.428	1.503		1.441	1.443	1.570
		Rank	12	13	12		13	14	13		15	15	15		16	16	16

When the same amount of spatial displacements is applied, more errors are expected with model B compared to model A due to the steeper dose gradients at penumbra regions. While the gamma method does not show such difference the IOA values demonstrate it clearly (e.g., 1.037 for model A vs. 1.081 for model B with intended 0% & 3 mm error in Table 2 and 1.318 vs. 1.404 in Table 3). Although model C has the same dose gradients as model A in penumbra regions, there is no flat region in model C unlike model A. Thus, model C is expected to have slightly larger errors with spatial displacements compared to model A. Such expectation can be observed in the IOA values but not in the gamma method (e.g., 1.037 for model A vs. 1.047 for model C with intended 0% & 3 mm error in Table 2 and 1.318 vs. 1.411 in Table 3). Because of such ability of QA result differentiation, the IOA method made it possible to place all the cases in order of overall uncertainty in each model (see the ranks from 1 to 16 indicated in Tables 2 and 3). Also note that these ranks are totally independent of the gamma acceptance criterion.

### 2-D systematic study using clinical cases

Tables 4 and 5 show the calculation results of the proposed indices (i.e., IOA, IOH and IOC values) and gamma evaluation (i.e., pass rate) under 1%/1 mm. 2%/2 mm, 3%/3 mm and 4%/4 mm criteria for one of head & neck cases, which used global and local normalization, respectively. In each example, dose errors ranged from − 3 to + 3% (of the maximum in the reference) and spatial displacements did from − 3 mm to + 3 mm in the lateral direction, resulting in a total of 48 erroneous situations. As can be seen, the values of IOA, IOH and IOC showed noticeable and reasonable variations from case to case, implying that the proposed index system was capable of differentiating QA results. It is worth to note that the IOA values are symmetric between the same magnitude of positive and negative dose errors (e.g., + 3% vs. -3% intended error). This can be easily expected from the definition of IOA. However, both the IOH and IOC values varied asymmetrically and provided additional information to decide whether the measured dose was hot or cold. In Table 4, the smallest IOC was 0.936 (with − 3%/− 3 mm intended error) and the largest IOH was 1.054 (with + 3%/+ 3 mm intended error). The IOA values at those two largest intended error situations were 1.06 and 1.059, respectively. The gamma pass rate became significantly low with large errors (i.e., when at least 3% dose error or 3 mm displacement error was involved) and reached the minimum of 66.4% under 3%/3 mm criteria in the case of − 3%/+ 3 mm intended error. However, it stayed 100% in 28 out of 48 situations, indicating that its capability of differentiating QA results significantly depended on acceptance criteria in many situations.

**Table 4:** Example of the global calculation results of the gamma evaluation and the index system for one of the head and neck cases applied spatial displacement along lateral direction

				Intentional Dose Error						
				−3%	−2%	−1%	0	+ 1%	+ 2%	+ 3%
Intentional Spatial Error	-3 mm	Gamma pass rate (%)	4%/4 mm	92.7	100.0	100.0	100.0	100.0	100.0	92.6
			3%/3 mm	69.9	81.5	88.6	100.0	89.2	82.6	69.5
			2%/2 mm	45.7	55.4	63.5	66.1	62.7	53.0	43.5
			1%/1 mm	13.3	17.4	22.2	23.3	20.2	15.9	12.1
		IOA		1.060	1.056	1.052	1.051	1.052	1.055	1.059
		IOH		1.022	1.025	1.029	1.034	1.039	1.045	1.052
		IOC		0.936	0.942	0.948	0.953	0.958	0.962	0.966
	-2 mm	Gamma pass rate (%)	4%/4 mm	100.0	100.0	100.0	100.0	100.0	100.0	100.0
			3%/3 mm	79.4	100.0	100.0	100.0	100.0	100.0	76.9
			2%/2 mm	47.6	65.4	79.4	100.0	78.8	62.6	46.9
			1%/1 mm	11.8	17.5	29.0	35.1	27.1	17.0	11.4
		IOA		1.049	1.043	1.039	1.038	1.039	1.042	1.048
		IOH		1.014	1.017	1.020	1.024	1.030	1.036	1.044
		IOC		0.948	0.955	0.961	0.966	0.971	0.975	0.978
	-1 mm	Gamma pass rate (%)	4%/4 mm	100.0	100.0	100.0	100.0	100.0	100.0	100.0
			3%/3 mm	94.6	100.0	100.0	100.0	100.0	100.0	92.9
			2%/2 mm	47.6	81.4	100.0	100.0	100.0	79.1	47.0
			1%/1 mm	8.2	16.1	40.7	100.0	37.2	16.0	7.4
		IOA		1.036	1.029	1.023	1.020	1.022	1.028	1.036
		IOH		1.005	1.007	1.009	1.013	1.019	1.026	1.035
		IOC		0.962	0.970	0.976	0.982	0.986	0.989	0.992
	0	Gamma pass rate (%)	4%/4 mm	100.0	100.0	100.0	100.0	100.0	100.0	100.0
			3%/3 mm	100.0	100.0	100.0	100.0	100.0	100.0	100.0
			2%/2 mm	55.3	100.0	100.0	100.0	100.0	100.0	55.8
			1%/1 mm	0	0.1	100.0	100.0	100.0	0.1	0
		IOA		1.030	1.020	1.010	1.000	1.010	1.020	1.030
		IOH		1.000	1.000	1.000	1.000	1.010	1.020	1.030
		IOC		0.970	0.980	0.990	1.000	1.000	1.000	1.000
	+ 1 mm	Gamma pass rate (%)	4%/4 mm	100.0	100.0	100.0	100.0	100.0	100.0	100.0
			3%/3 mm	92.8	100.0	100.0	100.0	100.0	100.0	95.0
			2%/2 mm	45.7	78.4	100.0	100.0	100.0	82.2	48.5
			1%/1 mm	7.1	15.4	36.5	100.0	41.3	16.6	8.4
		IOA		1.036	1.029	1.023	1.020	1.023	1.028	1.036
		IOH		1.005	1.007	1.010	1.014	1.019	1.027	1.035
		IOC		0.962	0.971	0.978	0.984	0.989	0.992	0.995
	+ 2 mm	Gamma pass rate (%)	4%/4 mm	100.0	100.0	100.0	100.0	100.0	100.0	100.0
			3%/3 mm	75.6	100.0	100.0	100.0	100.0	100.0	81.6
			2%/2 mm	44.3	60.7	78.5	100.0	79.9	67.0	49.9
			1%/1 mm	10.6	16.3	26.3	35.1	29.8	18.2	12.5
		IOA		1.049	1.043	1.039	1.038	1.039	1.042	1.048
		IOH		1.015	1.018	1.022	1.026	1.031	1.037	1.045
		IOC		0.950	0.958	0.964	0.970	0.976	0.980	0.984
	+ 3 mm	Gamma pass rate (%)	4%/4 mm	91.5	100.0	100.0	100.0	100.0	100.0	94.3
			3%/3 mm	66.4	80.1	88.1	100.0	89.9	84.4	74.1
			2%/2 mm	41.4	51.1	61.7	66.0	64.6	57.6	47.9
			1%/1 mm	11.1	14.9	19.5	23.3	23.0	18.2	14.1
		IOA		1.060	1.056	1.053	1.051	1.052	1.055	1.059
		IOH		1.024	1.028	1.032	1.036	1.041	1.047	1.054
		IOC		0.940	0.947	0.954	0.960	0.966	0.970	0.975

**Table 5:** Example of the local calculation results of the gamma evaluation and the index system for one of the head and neck cases applied spatial displacement along lateral direction

				Intentional Dose Error						
				-3%	-2%	-1%	0	+ 1%	+ 2%	+ 3%
Intentional Spatial Error	-3 mm	Gamma pass rate (%)	4%/4 mm	71.1	83.6	95.2	100.0	94.2	81.3	68.6
			3%/3 mm	52.8	63.7	73.0	100.0	72.7	62.6	52.0
			2%/2 mm	30.8	37.5	45.1	45.9	42.1	35.1	28.8
			1%/1 mm	6.0	6.9	9.0	8.0	8.0	5.7	4.8
		IOA		1.210	1.189	1.176	1.171	1.177	1.191	1.212
		IOH		1.083	1.095	1.109	1.127	1.148	1.173	1.202
		IOC		0.807	0.836	0.863	0.885	0.904	0.920	0.933
	-2 mm	Gamma pass rate (%)	4%/4 mm	79.5	90.9	98.2	100.0	98.0	89.1	76.5
			3%/3 mm	56.2	72.9	90.8	100.0	89.4	70.3	54.5
			2%/2 mm	30.8	41.9	56.6	100.0	54.3	41.3	30.3
			1%/1 mm	4.9	6.8	11.1	12.3	10.0	6.5	4.8
		IOA		1.175	1.148	1.130	1.124	1.131	1.149	1.176
		IOH		1.052	1.062	1.075	1.091	1.112	1.139	1.170
		IOC		0.833	0.865	0.893	0.916	0.933	0.946	0.957
	-1 mm	Gamma pass rate (%)	4%/4 mm	82.7	93.2	99.1	100.0	99.0	91.9	80.2
			3%/3 mm	63.4	81.4	95.6	100.0	95.1	79.8	61.7
			2%/2 mm	26.6	44.6	74.1	100.0	72.2	44.0	26.6
			1%/1 mm	3.6	6.5	14.6	100.0	13.7	6.5	3.3
		IOA		1.140	1.106	1.078	1.067	1.078	1.106	1.141
		IOH		1.018	1.024	1.033	1.048	1.071	1.104	1.140
		IOC		0.861	0.897	0.929	0.954	0.969	0.978	0.985
	0	Gamma pass rate (%)	4%/4 mm	83.1	93.4	99.3	100.0	99.3	93.1	81.8
			3%/3 mm	64.5	82.7	96.3	100.0	96.2	82.5	64.5
			2%/2 mm	28.0	50.5	79.9	100.0	80.2	51.4	29.0
			1%/1 mm	0	0	0.1	100.0	0.1	0	0
		IOA		1.124	1.083	1.041	1.000	1.041	1.083	1.124
		IOH		1.000	1.000	1.000	1.000	1.041	1.083	1.124
		IOC		0.876	0.917	0.959	1.000	1.000	1.000	1.000
	+ 1 mm	Gamma pass rate (%)	4%/4 mm	81.7	92.4	99.1	100.0	99.1	93.1	81.8
			3%/3 mm	61.7	80.1	95.1	100.0	95.5	81.5	63.4
			2%/2 mm	24.9	42.8	72.1	100.0	74.0	45.5	28.1
			1%/1 mm	3.1	6.1	13.3	100.0	14.9	6.8	3.9
		IOA		1.140	1.106	1.078	1.067	1.079	1.107	1.141
		IOH		1.018	1.025	1.034	1.049	1.072	1.105	1.141
		IOC		0.861	0.897	0.930	0.954	0.969	0.978	0.985
	+ 2 mm	Gamma pass rate (%)	4%/4 mm	77.7	89.7	98.2	100.0	98.3	91.1	79.7
			3%/3 mm	53.6	69.8	89.7	100.0	90.9	74.5	57.9
			2%/2 mm	27.8	39.1	53.5	100.0	57.7	43.8	32.9
			1%/1 mm	4.4	6.2	9.6	12.3	11.5	7.3	5.3
		IOA		1.174	1.149	1.131	1.125	1.132	1.151	1.178
		IOH		1.054	1.064	1.077	1.093	1.115	1.141	1.172
		IOC		0.834	0.866	0.894	0.917	0.934	0.947	0.957
	+ 3 mm	Gamma pass rate (%)	4%/4 mm	67.8	81.0	94.6	100.0	95.7	84.6	73.5
			3%/3 mm	50.1	60.1	71.6	100.0	75.0	67.3	55.8
			2%/2 mm	27.5	34.1	41.6	46.5	45.7	38.8	32.1
			1%/1 mm	4.4	5.3	7.7	8.0	9.4	7.3	6.4
		IOA		1.211	1.191	1.178	1.174	1.179	1.194	1.216
		IOH		1.087	1.099	1.114	1.131	1.152	1.177	1.205
		IOC		0.808	0.837	0.864	0.886	0.905	0.921	0.934

**Fig. 3:**
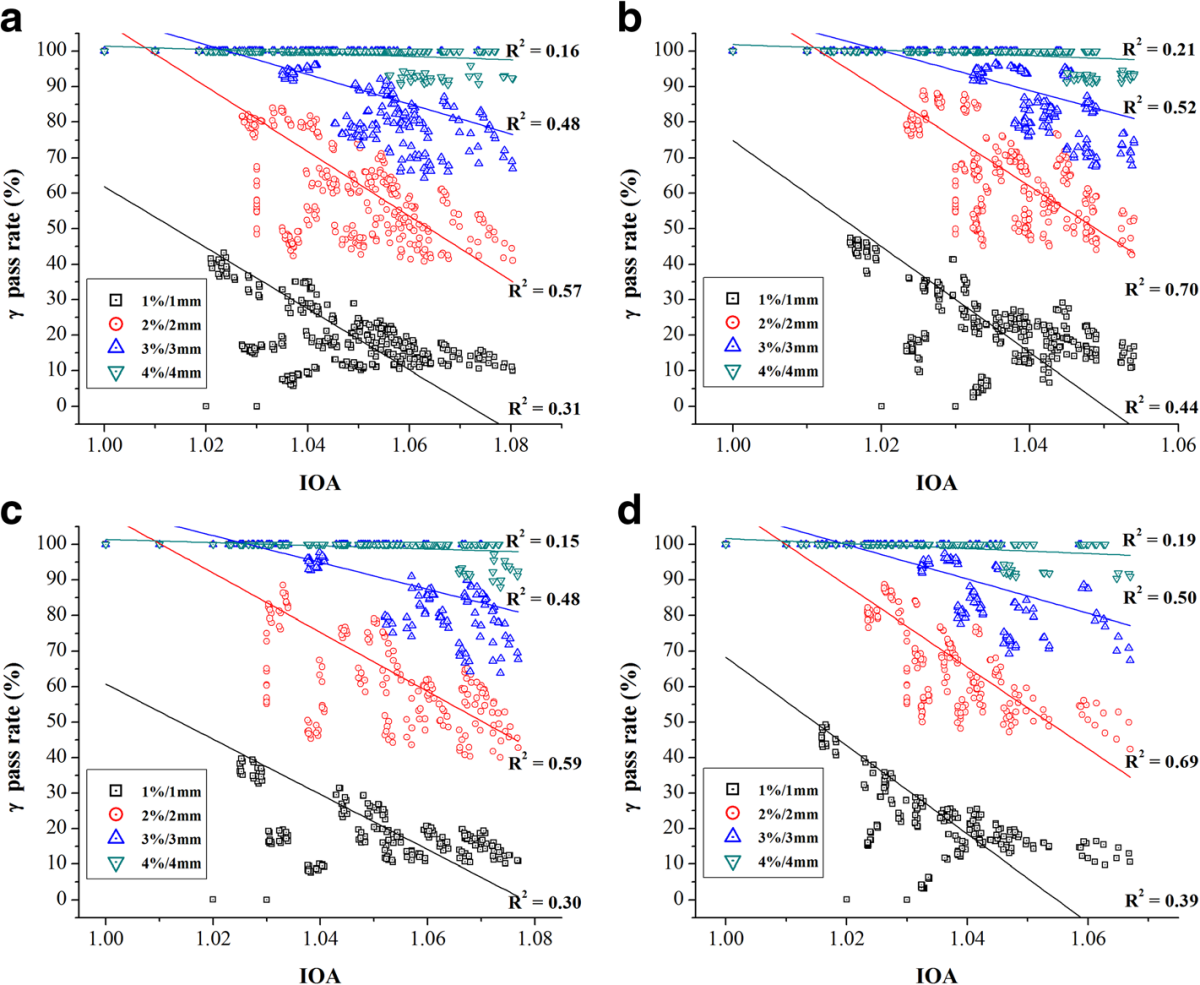
Scatter plots of relationship between the IOA and the gamma pass rate (%) for the global normalization with regard to direction of displacement: for 8 head and neck cases along (**a**) the lateral and (**b**) the longitudinal directions, and 5 prostate cases along (**c**) the lateral and (**d**) the longitudinal directions

**Fig. 4:**
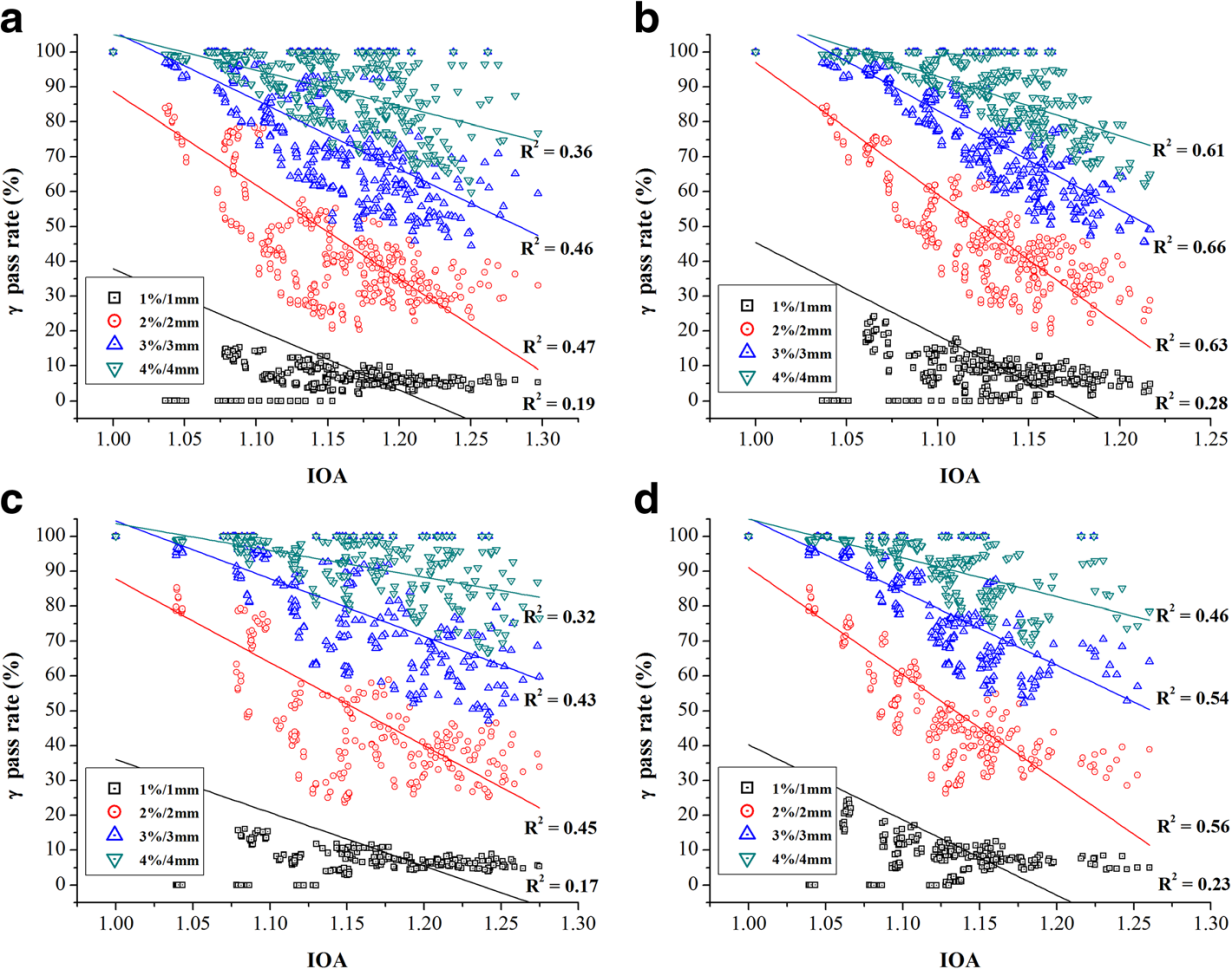
Scatter plots of relationship between the IOA and the gamma pass rate (%) for the local normalization with regard to direction of displacement: for 8 head and neck cases along (**a**) the lateral and (**b**) the longitudinal directions, and 5 prostate cases along (**c**) the lateral and (**d**) the longitudinal directions

Figures 3 and 4 show the scatter plots of the IOA values vs. the gamma pass rates in 4 different DD/DTA acceptance criteria (i.e., 1%/1 mm, 2%/2 mm, 3%/3 mm and 4%/4 mm), which used global and local normalization, respectively. Top 2 plots [i.e., (a) and (b)] are for 8 head & neck cases and bottom 2 plots [i.e., (c) and (d)] for 5 prostate cases. Left side [i.e., (a) and (c)] is for lateral displacements and right side [i.e., (b) and (d)] for longitudinal displacements. Solid lines indicate the trend between the IOA values and the gamma pass rates. In every case, the gamma pass rates tended to decrease as the IOA values did increase. Although such correlation seemed stronger under tighter gamma evaluation criteria in general, the highest correlation was obtained under the DD/DTA criterion of 2%/2 mm based on the regression analysis (R-square, *p* < 0.01).

### Application to compare calculated with measured data for clinical cases

**Fig. 5:**
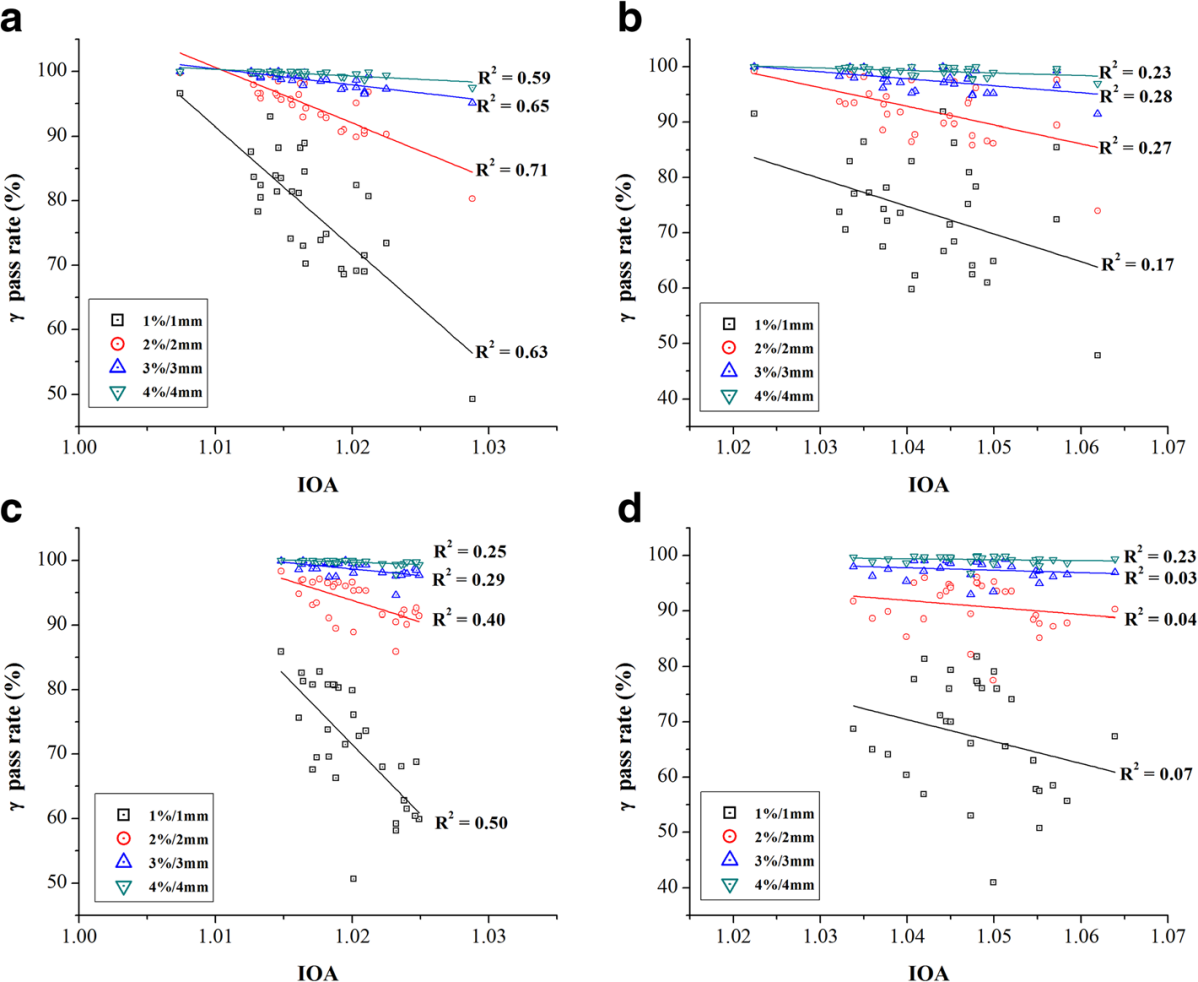
Scatter plots of relationship between the IOA and the gamma pass rate (%) for applying actual clinical VMAT QA cases (i.e., compared calculated with measured data): for 30 head & neck cases along (**a**) the global and (**b**) the local normalizations, and 30 prostate cases along (**c**) the global and (**d**) the local normalizations

Figure 5 shows the scatter plots of the IOA values vs. the gamma pass rates in 4 different DD/DTA acceptance criteria (i.e., 1%/1 mm, 2%/2 mm, 3%/3 mm and 4%/4 mm), which used global and local normalization, respectively (p < 0.01). Top 2 plots [i.e., (a) and (b)] are for 30 cases of head & neck and bottom 2 plots [i.e., (c) and (d)] for prostate cases, respectively. Left side [i.e., (a) and (c)] is for global normalization and right side [i.e., (b) and (d)] for local normalization. Solid lines indicate the trend between the IOA values and the gamma pass rates. In every case of gamma criteria, the gamma pass rates tended to decrease as the IOA values did increase. However, a correlation between IOA and gamma pass rate under 3%/3 mm and 4%/4 mm criteria seemed relatively weak, which was understandable. In Fig. 5 (a and c), we note IOA values were smaller than 1.03 in all of cases used in this study. With such results, it is not unreasonable to estimate that the overall global dose uncertainty was less than 3% in every case.

## Discussion

The proposed single index method is quite simple and intuitively easy to implement as an additional tool in IMRT QA for evaluating differences between planned and measured dose distribution. The proposed index system is fully based on point-by-point comparison and deals with dose difference directly. In fact, the quantity that is directly relevant to clinical outcome is ‘dose’ thus, spatial uncertainty itself (e.g., DTA) is incomplete to provide direct information necessary and it needs to be converted to ‘dose uncertainty’ to be more meaningful. Therefore, any approach including the gamma method that utilizes spatial information directly without conversion to dose information is subject to such limitation. The proposed IOA, IOH, and IOC are not intended to replace the existing gamma evaluation methods. However, it would be useful to estimate a range of index values which is reasonably acceptable in common practice. In case of global normalization, it was found in Fig. 5 (a and c) that most cases (i.e., 57 out of 60) having the IOA value of less than 1.025 showed 90% or higher pass rate under the 2%/2 mm global gamma test. Based on such observation, the value of 1.025 could be a good reference. Note, in principle, 1.025 implies that the overall dose difference of a plan is about 2.5%. Considering the definition (i.e., index of achievement), it would make more sense to use local normalization only in IOA calculation. However, it is common to use global normalization in IMRT QA thus, we included global normalization as well. Therefore, using the IOA, it is possible to figure out dose difference either absolutely based on a reference value or relatively by each point.

For qualitative assessment of IMRT QA, the pass rate based gamma evaluation method has been widely adopted as an essential technique in clinical practice and its application has been expanded from simple 2-D to 3-D and even to 4-D [17–24]. Recently, however, several publications have been made to report limitations of the gamma method [10, 25–32]. As illustrated in Tables 2 and 3, for example, it does suffer from lack of the ability of finely differentiating plans in terms of their quality depending on how the acceptance criterion is chosen. In comparison of the results between two model dose distributions, A with less steep penumbra and B with steeper penumbra, while it was possible to tell the difference between them using the index system by observing that relatively large difference of IOA values existed in B, it was not easy to do using the gamma method because the gamma pass rates were same in many situations. Similar behavior can be observed in Figs. 3 and 4. The scatter plots basically do not take a continuous trend from IOA = 1.0. Instead, they initially have points having a 100% pass rate until certain IOA value specific to given DD/DTA tolerance criterion then suddenly show points at lower pass rates and take continuous trend from there. Obviously, such non-continuous regions are range where the gamma method is insensitive for finely discerning QA results. Regardless of what the DD/DTA criterion is used, the index system provides the same values of IOA, IOH and IOC. In addition, those values are proportional to the amount of errors. In Figs. 3 and 4, in addition, the IOA not only showed relatively robust correlations with the gamma pass rates in certain range but also illustrated the possibility that it could complement the inexplicable part by the pass rate of the gamma analysis. Therefore, we believe the proposed index system can add value to the current gamma method by providing information that is often lost due to the acceptance criteria approach.

**Fig. 6:**
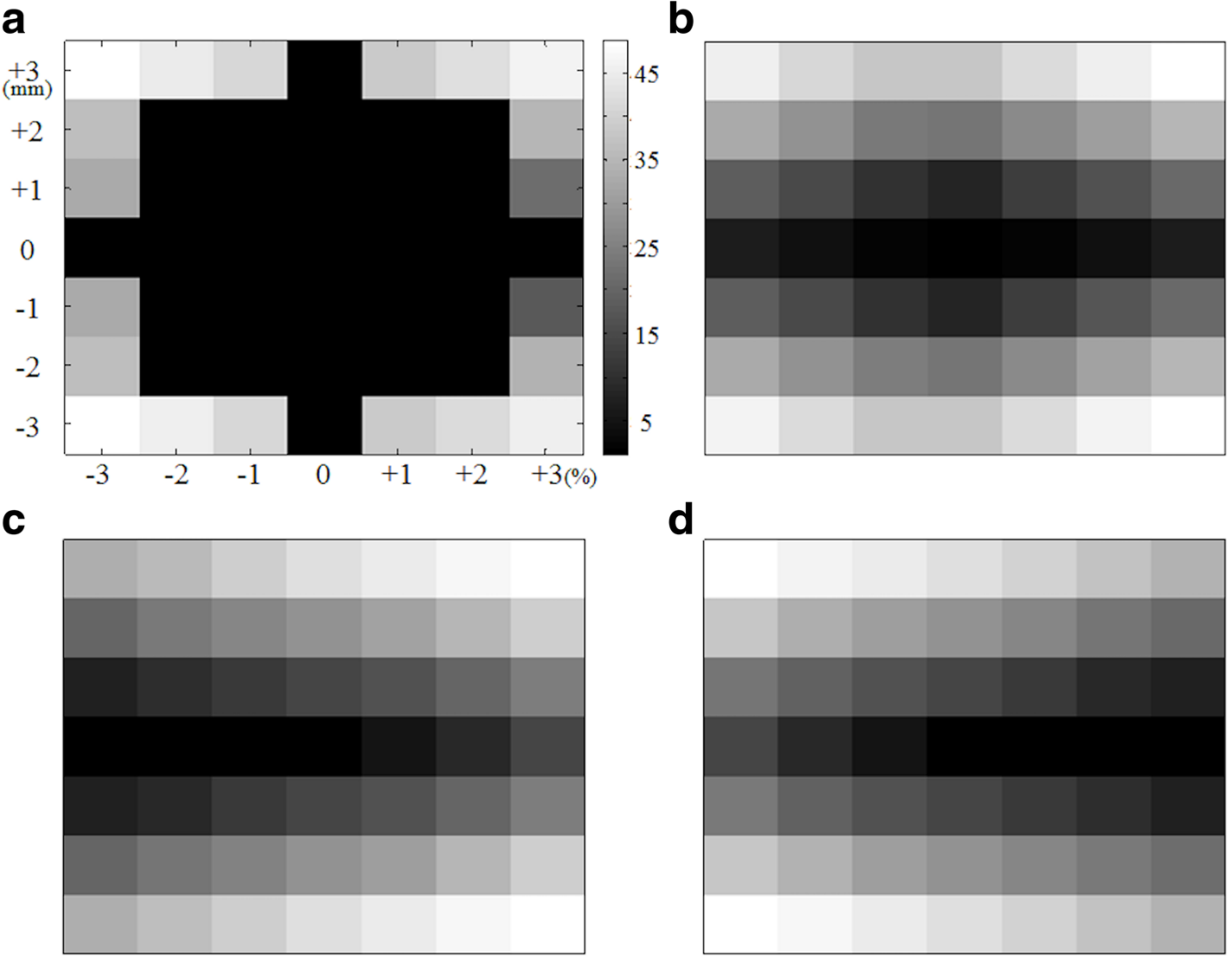
Example of rank maps for all of head and neck cases: (**a**) 3%/3 mm gamma evaluation, (**b**) IOA, (**c**) IOH, and (**d**) IOC for the global normalization. It consists of average rank from QA results with regard to each amount of dose difference from − 3 to + 3% by 1% intervals along lateral axis and/or spatial displacement by 1 mm intervals along longitudinal axis. Note that no change of levels among results indicates insensitivity, which means undistinguishable which plan is better

**Fig. 7:**
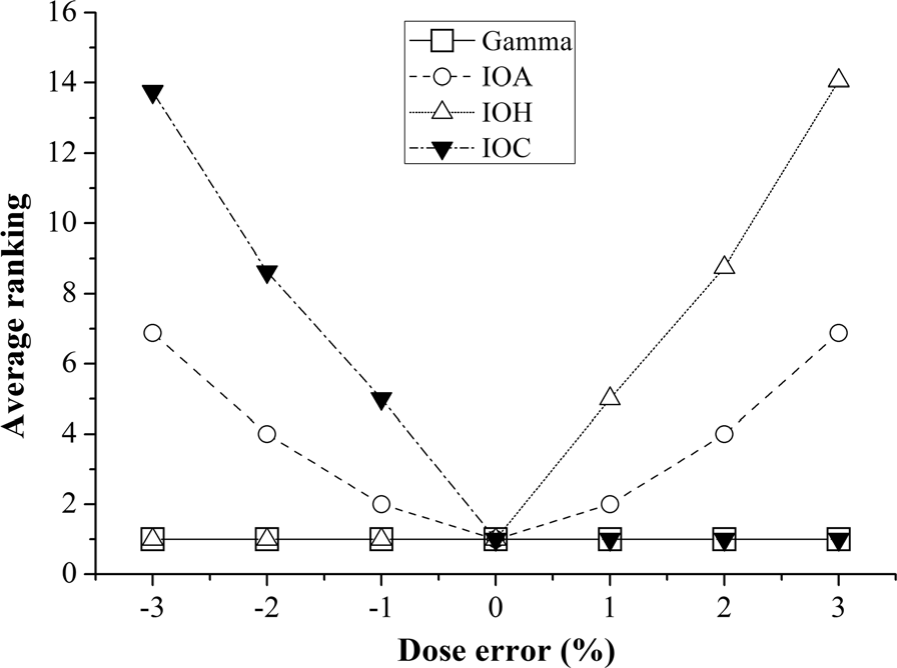
Line profiles measured along the non-displacement results in Fig. 6 (i.e., ‘0 mm’)

Figure 6 shows the average rank maps of QA results based on the (a) gamma pass rate under the criterion of 3%/3 mm, (b) IOA, (c) IOH and (d) IOC from all of head and neck cases. As described above, the gamma method obtained 100% pass rate in 28 out of 48 situations and was subject to insensitivity of QA result evaluation (since 28 cases got ranked with ‘1’ and were not distinguishable). However, the values of indices were more sensitive and enabled putting ranks in more detailed steps. Figure 7 shows ranking profiles measured along the ‘0 mm’ displacement line in Fig. 6 (a-d). As can be expected easily, the IOH and IOC values provided rank maps properly in terms of overdose (i.e., hotness) and underdose (i.e., coldness), respectively. In Fig. 6, it is worth to note that the values of rank vary more abruptly following the y-axis (i.e., the axis of intended spatial error) than x-axis (i.e., the axis of intended dose error). This trend indicates that spatial displacement has more impact on QA result than dose perturbation in the case studied. However, in general, such difference of importance is often not fairly taken into account in gamma method. When a DD/DTA criterion is chosen of 2%/2 mm, for example, regardless of their true importance both 1% dose error and 1 mm spatial error are considered to be same in their contribution to gamma value calculation by the definition of gamma. This, we believe, is the most serious limitation of the gamma method and the proposed index system in this study is able to compensate it to certain extent.

Recently, Steers et al. reported that the optimal acceptance criterion in arbitrary situations is closely related with the selected dose threshold in a gamma analysis [33]. Thus, it would be useful to systematically investigate characteristics of the proposed indices according to the level of dose threshold in addition to other variables such as acceptance criteria, dose distribution grid size and interpolation method.

A collapsed dose matrix is obtained in the case of Portal Dosimetry-based QA for VMAT. Obviously, such collapsed dose matrix is not able to mimic actual dose delivery and it cannot be considered ‘real’. However, it is a limitation of current Portal Dosimetry-based QA method in terms of ‘what to evaluate’ but not for ‘how to evaluate’. In other words, the proposed method is rather about ‘how to evaluate’ than ‘what to evaluate’ and the proposed method has nothing to do with which QA technique is used. From the view of index calculation, for instance, there is no difference between a realistic static-beam dose matrix and a collapsed dose matrix.

## Conclusions

We have proposed adding an index system to the current IMRT QA process for better understanding the result of IMRT QA and performed a systematic simulation study to evaluate the feasibility of the method proposed. The simulation study containing both hypothetical 1-D and clinical 2-D dose distributions demonstrated that the method was able to provide indices that were independent of acceptance criteria and enabled evaluating the matching quality of each plan with measurement.

Based on the findings, independency on acceptance criteria of the method will also help making clear communications among readers of published articles and researchers in multi-institutional studies. We believe this method can compensate some of limitations of the gamma-based QA method by providing valuable information that is often lost in the current approach.

## Abbreviations

1-D: One-dimensional
2-D: Two-dimensional
3-D: Three-dimensional
4-D: Four-dimensional
DD: Dose difference
DTA: Distance to agreement
EPID: electronic portal imaging device
IMRT: Intensity modulated radiation therapy
IOA: Index of achievement
IOC: Index of coldness
IOH: Index of hotness
IRB: Institutional review board
OAR: Organ at risk
PDIP: portal dose image prediction
QA: Quality assurance
ROI: Region of interest
VMAT: Volumetric modulated arc therapy
